# The Pivotal Role of Adipocyte-Na K peptide in Reversing Systemic Inflammation in Obesity and COVID-19 in the Development of Heart Failure

**DOI:** 10.3390/antiox9111129

**Published:** 2020-11-14

**Authors:** Zi-jian Xie, Joel Novograd, Yaakov Itzkowitz, Ariel Sher, Yosef D. Buchen, Komal Sodhi, Nader G. Abraham, Joseph I. Shapiro

**Affiliations:** 1 Joan C. Edwards School of Medicine, Marshall University, Huntington, WV 25755, USA; xiez@marshall.edu (Z.-j.X.); sodhi@marshall.edu (K.S.); 2 Department of Medicine, New York Medical College, Valhalla, NY 10595, USA; jnovogra@student.nymc.edu (J.N.); yitzkowitz90@gmail.com (Y.I.); asher@student.touro.edu (A.S.); ybuchen@student.nymc.edu (Y.D.B.)

**Keywords:** Na/K-ATPase, reactive oxygen species, coronavirus disease, heme oxygenase, obesity, adipocytes, heart failure

## Abstract

This review summarizes data from several laboratories that have demonstrated a role of the Na/K-ATPase, specifically its α1 subunit, in the generation of reactive oxygen species (ROS) via the negative regulator of Src. Together with Src and other signaling proteins, the Na/K-ATPase forms an oxidant amplification loop (NKAL), amplifies ROS, and participates in cytokines storm in obesity. The development of a peptide fragment of the α1 subunit, NaKtide, has been shown to negatively regulate Src. Several groups showed that the systemic administration of the cell permeable modification of NaKtide (pNaKtide) or its selective delivery to fat tissue—adipocyte specific expression of NaKtide—ameliorate the systemic elevation of inflammatory cytokines seen in chronic obesity. Severe acute respiratory syndrome – coronavirus 2 (SARS-CoV-2), the RNA Coronavirus responsible for the COVID-19 global pandemic, invades cells via the angiotensin converting enzyme 2 (ACE-2) receptor (ACE2R) that is appended in inflamed fat tissue and exacerbates the formation of the cytokines storm. Both obesity and heart and renal failure are well known risks for adverse outcomes in patients infected with COVID-19. White adipocytes express ACE-2 receptors in high concentration, especially in obese patients. Once the virus invades the white adipocyte cell, it creates a COVID19–porphyrin complex which degrades and produces free porphyrin and iron and increases ROS. The increased formation of ROS and activation of the NKAL results in a further potentiated formation of ROS production, and ultimately, adipocyte generation of more inflammatory mediators, leading to systemic cytokines storm and heart failure. Moreover, chronic obesity also results in the reduction of antioxidant genes such as heme oxygenase-1 (HO-1), increasing adipocyte susceptibility to ROS and cytokines. It is the systemic inflammation and cytokine storm which is responsible for many of the adverse outcomes seen with COVID-19 infections in obese subjects, leading to heart failure and death. This review will also describe the potential antioxidant drugs and role of NaKtide and their demonstrated antioxidant effect used as a major strategy for improving obesity and epicardial fat mediated heart failure in the context of the COVID pandemic.

## 1. Introduction

### 1.1. COVID Infection and Obesity

SARS-CoV-2, the virus responsible for COVID-19, is causing a worldwide pandemic that has currently infected over 16 million people with more than 600,000 deaths. In some individuals, SARS-CoV-2 infection induces a severe inflammatory cytokine storm, resulting in high rates of morbidity and mortality. Common symptoms of COVID-19 include dry cough, fever, myalgia, difficulty breathing, fatigue, and milder symptoms, including nausea, vomiting, headaches, and loss of taste and smell [1]. Certain underlying conditions have been shown to increase the risk of COVID-19 infection and the severity of its adverse effects. These conditions include respiratory problems, heart disease, immunocompromised states, severe obesity (body mass index (BMI) of 40 or higher), diabetes, chronic kidney disease, and liver disease [2]. SARS-CoV-2 is an RNA beta coronavirus that is a subgenus of the SARs virus family. The SARS-CoV-2 virus enters cells that express ACE2R on their surface [3,4,5,6]. During the entry process, the virus binds not only ACE2 receptor, but also porphyrin by means of the so called spike protein on the virus capside [7]. This leads to increased levels of free heme and a reduction in functional hemoprotein, with a resultant increase in inflammatory reactions. During COVID-19 infection oxidation-induced carbonylation of the Na/K-ATPase α1 subunit, which desensitizes the Na/K-ATPase signaling, results in increased ROS and inflammation which can further promote the inflammatory cytokine storm that characterizes the course of complicated COVID-19 infection. Since the α1 subunit is a negative regulator of Src, its carbonylation leads to increased Src activity and ROS formation [8,9,10]. Blocking the adipocyte Na/K-ATPase oxidant amplification loop using adipocyte-specific NaKtide expression has been shown to improve uremia, reduce oxidant stress, reduce local elevation of inflammatory cytokines, and alleviate uremic cardiomyopathy [11].

COVID-19 has been shown to affect cells with both high and low ACE2R concentrations. However, significantly worse effects are seen in patients with high expression levels of ACE2 receptor [12]. Notably, obesity results in a higher number of white adipocytes compared to brown adipocytes, resulting in mitochondrial dysfunction, increased inflammation, and an increase in the ACE2 receptor levels. The signs and symptoms seen in patients with the novel coronavirus have been predominantly shown on organs such as the lungs, kidneys, and the heart, which all express ACE2R. Importantly, the redox state of adipocytes has been shown to regulate the progression of uremic cardiomyopathy in partially nephrectomized mice. This means that oxidative stress, caused by increased carbonylation of the Na/K-ATPase α1 subunit and the subsequent increased activity of Src, may contribute to renal failure and subsequent renal-cardiac syndrome [11].

Both COVID-19 and obesity have deep, adverse effects on cardiac function. Excess pericardial fat found in obesity increases inflammatory cytokines near the heart, similar to the inflammatory cytokine storm seen in COVID-19 infection [13,14,15]. Additionally, obese patients are susceptible to heart failure following COVID-19 infection due to the increase in inflammatory cytokines. COVID-19 morbidity and mortality are indeed higher in obese patients. Since white adipocytes have an upregulation of ACE2 receptor, obese patients provide an ideal scenario for higher COVID-19 entry into their cells. Through exploring the pathophysiological process in COVID-19, novel treatment options might be found while waiting for a viable vaccine.

Increased ROS and levels of inflammatory cytokines have also been implicated in the ebola virus (EBOV) [16,17]. Obesity and increased levels of circulating interleukin-6 (IL-6) have been associated to the morbidity and mortality seen in Human immunodeficiency virus (HIV) [18,19]. Inducing of antioxidant genes such HO-1 expression had similar effects in patients with HIV. Administration of Anti-HIV drug; AZT-Heme arginine increases antioxidant levels and inhibits viral RNA replication enzyme [20,21,22]. Additionally, like COVID-19, SARS/MERS infections have been associated with massive inflammatory cytokine storms [23,24]. A possible association, albeit a weak one, has also been suggested between decreased HO-1 expression and epidemics [25].

SARS-CoV-2 patients are known to have a severe systemic inflammatory reaction which includes degradation of heme to porphyrin and iron. The increased iron levels can overwhelm the cytoprotective capacity of HO-1, resulting in the formation of reactive oxygen species (ROS) [26] and augmented carbonylation of the Na/K-ATPase α1 subunit.

### 1.2. Obesity and Its Global Importance

Obesity is a major risk factor for non-communicable diseases including cardiovascular disease, diabetes, renal pathologies, musculoskeletal disorders, and some cancers [27]. Obesity can be measured using the BMI scale which measures the weight to height ratio (kg/m2). Although BMI does not directly measure body fat, research has shown that BMI correlates with other direct fat measurements, such as underwater weighing, dual energy x-ray absorptiometry, bioelectrical impedance, as well as skinfold thickness measurements [28,29,30]. A BMI greater than 25 is considered overweight, while that greater than 30 is considered obese. According to the World Health Organization, worldwide obesity has almost tripled since 1975 and more than 1.9 billion adults (18 years and older) in 2016 were considered overweight, of which 650 million were obese.

### 1.3. The Interplay between Obesity and the Na/K-ATPase Pump Can Provide an Inflammatory Platform That Exacerbates COVID-19 Infection

Obesity is a major risk factor for insulin resistance, vascular dysfunction, hypertension, and diabetes [26,31,32,33] and is also characterized for an increased level of circulating and tissue resident inflammatory cytokines and adipokines that have adverse effects on organ function and vasculature [34,35,36]. Adipose tissue in humans is located around internal organs (visceral fat), beneath the skin (subcutaneous fat), in bone marrow (yellow bone marrow), in breast tissue, and intramuscularly (muscular system), and it can be characterized as white, beige, or brown adipose tissue. Brown adipose tissue has high thermogenic activity due to higher number of mitochondria expressing uncoupling protein 1 (UCP-1). White adipose tissue consists of mature adipocytes that store fat resulting in further hyperplasia and hypertrophy. Generally, the white fat cell dysfunction in obesity is assumed to be a consequence of the pathogenesis of obesity, but studies have shown that the adipocyte plays a key role in the pathogenesis of obesity, creating the conditions for increased oxidative stress, leptin and insulin resistance, and promoting renal and cardiovascular disease [37]. White fat cells have lower density of insulin receptors and actively release tumor necrosis factor α (TNFα), IL-6, and adiponectin [34,38].

Interventions that diminish fatty acid accumulation, such as the arachidonic acid metabolite EET [39,40] or inducers of the antioxidant gene HO-1, e.g., cobalt compounds such as cobalt chloride (CoCl_2_) and cobalt protoporphyrin (CoPP) IX dichloride, are associated with increased insulin sensitivity, vascular function, improve cardiac function in heart failure patients, decreased levels of NOV, IL6 and TNF and the conversion of white adipocytes to brown adipocytes [32,41,42,43]. When administered to obese mice, herbal medicines with the ability to lower ROS, e.g., pomegranate seed oil or Thymoquinone present in black seed oil, reduce obesity-induced ROS Inflammation, NOV, and are accompanied with the conversion of white adipocytes to beige fat [44,45]. In obesity the white adipocyte phenotype can be induced by mitochondrial dysfunction, augmented ROS production, systemic oxidative stress, and an increase in inflammation [7,32,45,46,47]. However, it has been shown in a number of tissues and organs, including the kidney, adipose tissue, and heart, that Na/K-ATPase can exert the so-called oxidant amplification loop pathway (NKAL) [11,48,49,50,51]. This mechanism, initiated by ROS, results in further production of ROS downstream the NKAL pathway, where the activated Na/K-ATPase functions as a scaffolding protein [52,53]. Typically, the Na/K-ATPase α1 subunit negatively regulates Src activity; however, when an increased oxidant environment causes carbonylation of the α1 subunit of the pump, the pump becomes inactivated, allowing Src to promote additional ROS production [54,55].

**Figure 1:**
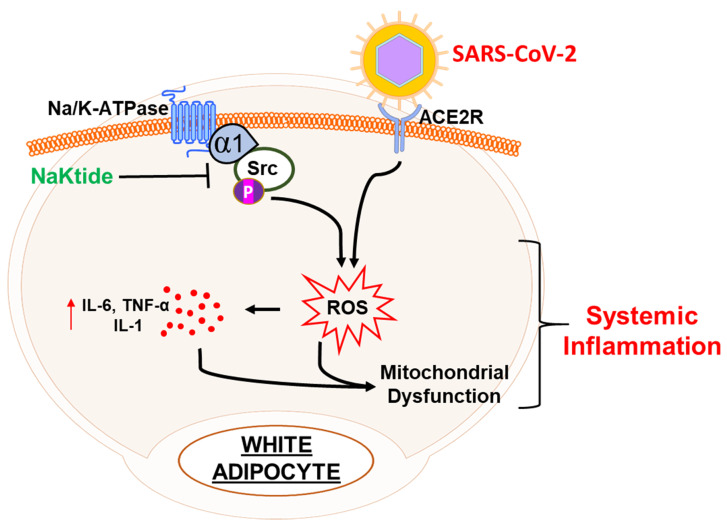
Adipocytes, NaKtide, and COVID-19 inflammation.

White adipocytes have increased carbonylation of the α1 subunit of Na/K-ATPase. There has been research showing that using an adipocyte-specific NaKtide, driven by the adiponectin promoter in a lentiviral vector, inhibits pathological Na/K-ATPase signaling and Src activation that results from α1 carbonylation [11,56]. In fact, synthetic pNaKtide has been shown to be effective in reducing obesity, oxidative stress, and cardiovascular disease [48]. Since obesity results in the increase in ROS and increase in inflammatory markers, this may have a negative effect on COVID-19 and the cytokine storm associated with it. It appears that using pNaKtide as a Src antagonist may reduce ROS and potentially ameliorate negative effects of COVID-19 (Figure 1). In this article, we will further discuss the ramifications of increased ROS due to the interplay between obesity and COVID-19 and how that can result in renal dysfunction and cardiomyopathy. We will discuss the use of NaKtide as potential therapeutic agents to ameliorate the adverse effects on the kidneys and heart in infected subjects with COVID-19.

In COVID-19, the viral spike protein binds weakly to the ACE2R but with strong affinity to the porphyrin ring [7]. Heme is made of porphyrin, thus allowing COVID-19 to access the cells via the ACE2 receptor and bind functional hemoprotein. The subsequently increased free heme levels, and decreased levels of functional hemoprotein and increased in NaKATPase can heavily contribute to COVID-19-induced systemic inflammation.

**Figure 2:**
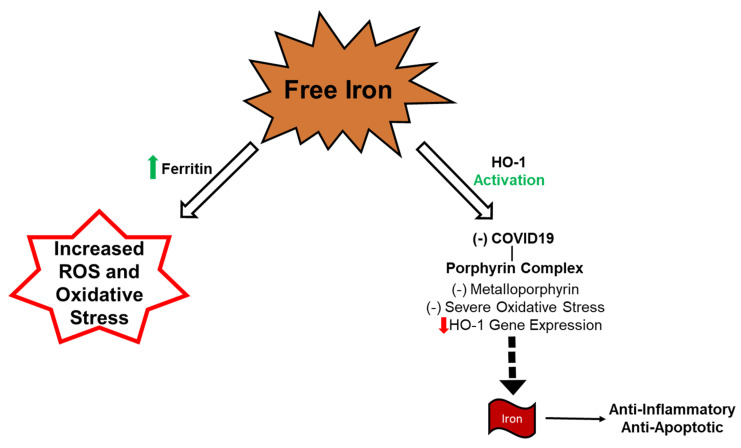
Free iron due to COVID-Porphyrin Complex increases ROS and oxidative stress.

Recent research suggests that altered iron homeostasis plays a role in COVID-19 pathogenesis [57]. COVID-porphyrin complexes release free iron producing increased ferritin synthesis. Additionally, the cytokine storm seen in COVID-19 also stimulates further ferritin synthesis [7]. This increase in ferritin causes an interaction between oxygen and free generating ROS [57]. Iron enters the cells through the regulator hepcidin reacting with ferroproteins [58]. Iron dysmetabolism may occur with ferroptosis and hyperserotonemia in hepcidin-like activity of COVID-19 [59]. This iron metabolism dysfunction can impair the transport of O2 contributing to the difficulty in breathing seen in COVID-19 infection [60]. Viruses have also been shown to depend on iron to replicate in host cells [61]. This increase in iron can further induce SARS-CoV-2 infection and reproduction in patients. Free iron has also shown damaging effects by iron toxicity in increasing ROS resulting in negative outcomes for the kidneys, liver, and heart. A lack of antioxidants such as NaKtide results in free heme and iron, causing inflammation from the bound COVID–porphyrin complex, as shown in Figure 2.

### 1.4. White Adipocyte vs Brown Adipocyte: Negative Implications in COVID-19

**Figure 3:**
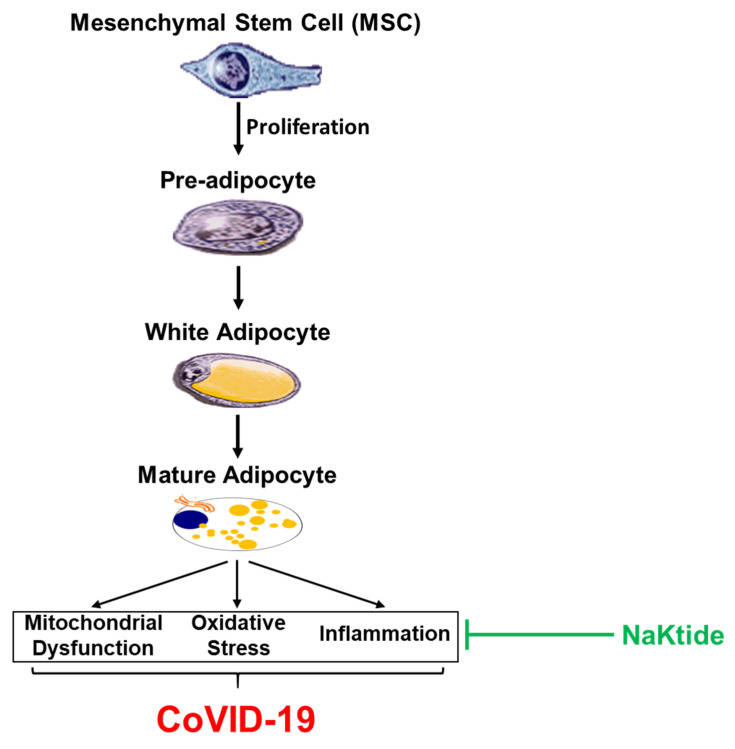
White adipocyte, Beige Adipocyte and Brown Adipocyte and COVID-19.

Obese individuals carry a higher amount of white adipose tissue when compared to lean individuals, who have a higher number of brown adipocytes. Additionally, white adipocytes, which are proinflammatory, have fewer mitochondria compared to brown fat cells. Brown adipocytes have increased intracellular droplets and UCP-1 that allow for non-shivering thermogenesis during cold stress. In contrast, white adipose tissue is unilocular, stores energy, and secretes adipokines [62], as shown in Figure 3. White adipocytes also present with mitochondrial dysfunction which can be ameliorated by increasing levels of peroxisome proliferator-activated receptor gamma coactivator 1-alpha (PGC1α), a regulator of mitochondrial biogenesis and adaptive thermogenesis. Additionally, increased levels of mitochondrial fusin proteins mitofusin 1 and 2 (MFN1 and MFN2) and Sirtuin 1 (SIRT1) are all responsible for the improvement of mitochondrial function and “browning” of white adipocytes [63]. Mitochondria levels are important as they produce a significant amount of ROS during the electron transport chain (ETC) [64]. During oxidative phosphorylation in the production of adenosine triphosphate (ATP), some electrons leak through complex I and III back into the mitochondrial matrix, binding to oxygen, forming superoxide anion or hydrogen peroxide. This superoxide anion may result in further formation of free radicals. In brown fat, UCP can exert a neutralization of the harmful ROS through the uncoupling of mitochondrial respiration, which causes protons to leak through the membrane, producing heat from the electrochemical energy. UCP can also be induced by carbon monoxide. Obese individuals have decreased brown fat and therefore decreased uncoupling and thermogenesis resulting in increased ROS. When diabetes and obesity co-exist, there are even more white adipocytes than brown adipocytes, and thus fewer mitochondria to perform thermogenesis.

It has been shown that the treatment of obese mice with adipocyte-specific NaKtide stimulates mesenchymal stem cells within adipose tissue and the committed pre-adipocyte progresses towards brown adipocyte linages. Mesenchymal stem cells serve as a reservoir in the formation of brown adipocyte by adipocyte specific NaKtide, not only ensuring sustained production of brown adipocytes but also the browning of other adipocytes and decreasing M1-like macrophage infiltration, reduced level of inflammatory molecules, and weight loss. Notably, the reduction of epicardial fat in humans and mice increases left ventricle function and prevents heart failure [13]. In the particular case of obese patients with COVID-19 infection, the effects of NaKtide on reducing cytokine storm represent a potential therapeutic benefit that could help to expedite the recovery of obese patients coursing with COVID-19, while we await more efficient antivirals and/or effective vaccines. Finally, the NaKtide targeting of adipose tissue, whether with tissue specific lentiviral vectors or through brown stem cell strategies, is also a promising strategy for the treatment of metabolic syndrome.

Cells expressing high levels of ACE2 receptor may have an increased intake of the novel SARS-CoV-2, resulting in a more severe infection and inflammation. White adipocytes express more ACE2 receptors than their counterparts, namely the beige and brown fat cells. This is in line with the more severe cases of COVID-19 infection and inflammation in obese patients, likely due to the upregulation of the ACE2 receptor on the white adipocyte cells.

### 1.5. Obesity and Oxidative Stress

Obesity has been shown to increase inflammatory cytokines and ROS, and vascular dysfunction and insulin resistance are increased [31,34,65,66]. It has been shown that mice fed a HFD have increased inflammatory cytokines IL-1, IL-6, and TNFα [13,32,67,68,69]. The activation of the angiotensin II system (Ang II) and nicotinamide adenine dinucleotide phosphate oxidase (NADPH oxidase) in obesity results in cardiovascular disease, hypertension, and diabetes [70,71]. These results are due to an increase in ROS induced by the RAS system which results in hypertrophy of adipocytes [72]. Additionally, obesity and diabetes play a role in the development of hyperglycemia resulting in high levels of ROS and heme, which work collectively to cause adipocyte and vascular dysfunction while suppressing HO-1 [73,74,75,76,77]. This activation of Ang II and NADPH oxidase in obesity, resulting in increased ROS, could also further exacerbate COVID-19 cytokine storm.

**Figure 4:**
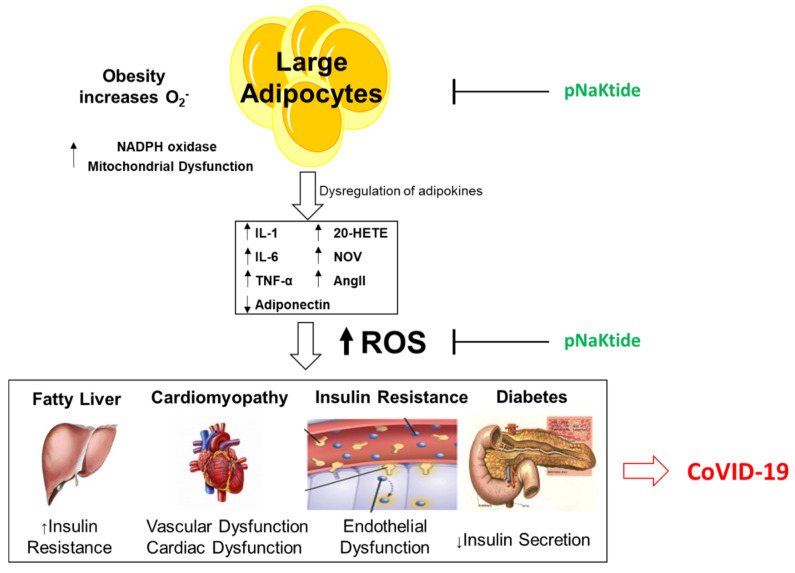
Obesity’s effect on cardiovascular disease, Fatty Liver, Cardiomyopathy and Diabetes exacerbating COVID-19.

It is well established that obesity greatly increases cardiovascular disease with clear negative effects on hypertension and atherosclerosis. Notably, non-alcoholic fatty liver disease (NAFLD) and its more advanced form, non-alcoholic steatohepatitis (NASH), are now recognized as independent factors in the development and progression of atherosclerosis, the main cause of coronary heart disease, and stroke. It is thus not surprising that the increased NADPH oxidase activity, heme levels, and mitochondrial dysfunction, together with increases in NaKATPase that are seen in obese individuals, strongly correlate with the vascular pathology observed in these patients [73], as illustrated in Figure 4. It is thus possible that the increased NADPH oxidase activity and suppression of antioxidants that takes place in obesity can further aggravate COVID-19 infection and some of its associated vascular complications.

**Figure 5:**
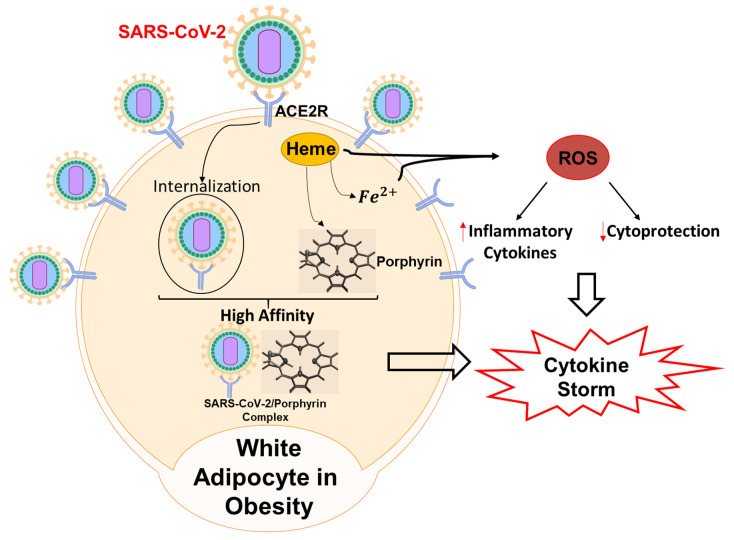
Obesity, COVID-19, ACE-2 receptor and the cytokine storm.

Patients with obesity and diabetes have a high baseline proinflammatory state which can worsen outcomes during SARS-CoV-2 infection. This proinflammatory state is a result of increased IL-6 from leptin and insulin resistance that takes place in obesity [78]. The resultant increased appetite further perpetuates the leptin and insulin resistance and IL-6 mediated inflammation. Obesity is also proinflammatory due to the increased levels of oxidized high-density lipoprotein (OX-HDL). Specifically, OX-HDL, a form of ROS, plays a role in adiposity and vascular dysfunction and further increases IL-1, IL-6, TNFα, and Ang II, potentiating the release of inflammatory cytokines by acting directly on mesenchymal stem cells. OX-HDL is also associated with an increase in the vasoconstrictor 20-HETE and correlates strongly with obesity-induced oxidative stress and endothelial dysfunction [79]. Therefore, OX-HDL can also further amplify the negative effects of COVID-19 increasing oxidative stress and further inducing a severe cytokine storm. Figure 5. A cytokine storm is a physiological reaction in which the innate immune system causes an uncontrolled release of signaling proinflammatory cytokine molecules [80]. During COVID-19 infection, this cytokine storm can result in multiple organ failure and mortality. Signs and symptoms of a cytokine storm include inflammation, nausea, fatigue, and a high fever.

The cytokine storm is known to nitric oxide synthetase (iNOS)-dependent production of peroxynitrite, a potent oxidant, from superoxide anions. Peroxynitrite exerts toxic effects on vascular endothelium. (Reviewed in [26,73]) Since iNOS and peroxynitrite levels are higher in obese patients, this can also make them more susceptible to the life-threatening effects of the cytokine storm that takes place during COVID-19 infection. This can be exacerbated by the accompanying increase in Src-induced ROS formation subsequent to carbonylation of the α1 subunit of Na/K-ATPase.

The increased oxidative stress seen in obesity and diabetes appears to play a role in the development of uremic cardiomyopathy [11]. Blocking adipocyte NKAL using adipocyte-specific NaKtide expression has been shown to reduce uremia, oxidant stress, the local elevation of inflammatory cytokines, and to ameliorate uremic cardiomyopathy [11]. Therefore, we speculate that using adipocyte-specific NaKtide and HO-1 inducers should have a beneficial effect in COVID-19 patients by ameliorating oxidative stress, especially in those at higher risk due to increased baseline inflammation, such as diabetic and/or obese patients.

### 1.6. pNaKtide as a Therapeutic Tool for Renal-Cardiomyopathy and Heart Failure

**Figure 6:**
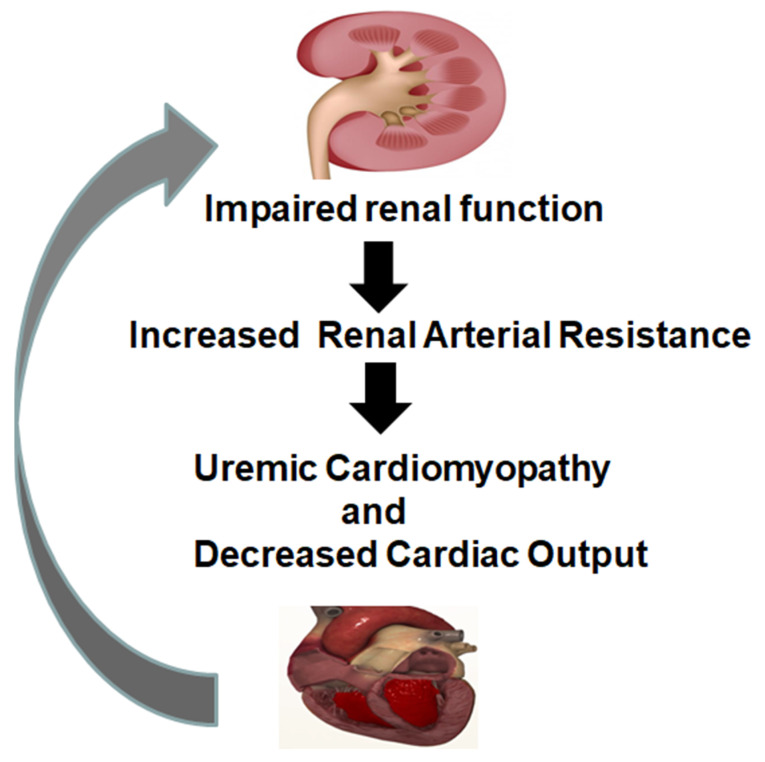
Link between Renal-Cardiomyopathy and heart failure.

A significant amount of experimental evidence supports the existence of a cardio-renal axis (Figure 6). Clinically, patients with renal dysfunction consistently course with cardiovascular dysfunction. This dysfunction has been termed uremic cardiomyopathy, which includes left ventricular diastolic dysfunction, left ventricular hypertrophy, and left ventricular systolic dysfunction [81]. Chronic kidney disease (CKD) is strongly associated with coronary artery disease, congestive heart failure, and cardiac arrest. Interestingly, CKD and CVD are both associated with inflammation and increased oxidative stress due to increased ROS [82]. Increased oxidative stress and mitochondrial dysfunction are both documented in patients with uremic cardiomyopathy [83].

As we mentioned previously, the inflammatory cytokine storm reported in COVID-19 is the result of increased levels of free heme and iron that cause the formation of ROS. The increased ROS induces carbonylation of the Na/K-ATPase α1 subunit exacerbating ROS production, mostly via Src, and inflammation, leading to NKAL in adipocytes. In obese patients who have increased levels of adipocytes with high ACE2 receptor concentrations, this can lead to severe inflammatory reactions and a positive, vicious feedback loop that results in further oxidative damage and inflammation.

In addition to the adverse effects of α1 carbonylation seen in adipocytes, Na/K-ATPase activity in the renal proximal tubule (RPT) is extremely susceptible to oxidative stress. Indeed, carbonylation of the Na/K-ATPase α1 subunit in the RPT has been shown to adversely affect the handling of sodium [84]. Specifically, α1 carbonylation results in pathological increase in urinary sodium excretion and has a desensitizing effect on Na/K-ATPase signaling and ultimately, salt sensitivity [85]. Notably, the redox state of adipocytes has been shown to regulate progression of uremic cardiomyopathy mice. Thus, oxidative stress caused by carbonylation of the Na/K-ATPase α1 subunit and the subsequent increased activity of Src may cause renal failure and subsequent renal-cardiac syndrome [11]. pNaKtide, a Src inhibitor, has been shown to effectively block the Na/K-ATPase oxidant amplification loop, improving the outcome and decreasing risk of a number of diseases associated to oxidative stress such as obesity, steatohepatitis, atherosclerosis, and cancer. pNaKtide has also been shown to improve outcomes in ischemia-reperfusion related heart disease and ameliorate pathological changes seen in uremic cardiomyopathy [49,86]. These positive effects were shown to increase with the dosage of pNaKtide. Studies demonstrated that treating HFD fed mice with pNaKtide resulted in less body weight gain decreased oxidative and inflammatory stress and increased insulin sensitivity, showing an effective amelioration and even reversion of the HFD effects. Thus, selective pNaKtide-mediated inhibition of the Na/K-ATPase oxidant amplification loop in fat tissue has been suggested as a possible therapy to treat and prevent obesity, insulin resistance, and metabolic syndrome [48]. Other investigators have shown that targeting fat tissue with antioxidant genes or inducer of antioxidants ameliorates renal dysfunction, improves mitochondrial function, and alleviates heart failure [87,88,89]. In mice fed a Western diet, adipocyte-specific NaKtide improves mitochondrial dysfunction by increasing levels of PGC1α which regulates mitochondrial biogenesis and adaptive thermogenesis. Adipocyte-specific expression of NaKtide also increased the levels of MFN1, MFN2, and SIRT1, all of which are responsible for improved mitochondrial function and the “browning” of fat [11,56]. These beneficial effects were positively correlated with a decrease in the α1 carbonylation of the Na/K-ATPase upon NaKtide treatment [90]. In the context of COVID-19 infection, this can result in further oxidative insult that contributes to heart failure in these patients.

### 1.7. NaKtide as Therapeutic Targets for Epicardial Fat and Heart Failure

**Figure 7:**
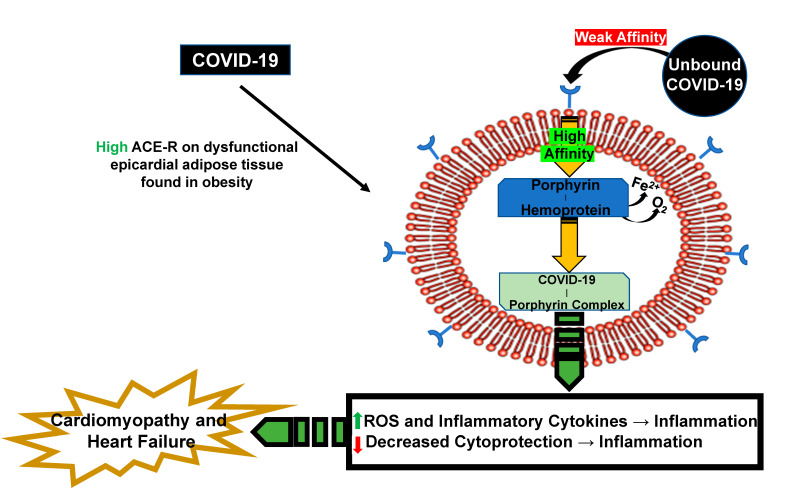
Schematic of increased ACE2R on white adipocytes in pericardial fat. There is increased baseline inflammation in obesity which is exacerbated due to COVID-19 infection. This can lead to subsequent cardiomyopathy and heart failure.

As discussed previously, COVID-19 induces a cytokine storm due to increased inflammatory cytokines and ROS, as well as reduced cytoprotection due to carbonylation of Na/K-ATPase and HO-1 deficiency. The virus invades cells via ACE2R and also binds porphyrin to form a COVID-porphyrin complex that releases free heme and iron contributing to the inflammatory response. This is particularly important in in organs displaying high levels of ACE2 receptor, such as the heart and kidneys. Additionally, the virus seems to have more severe effects on patients with obesity who already have higher baseline levels of inflammation. Obesity has more ACE2R on high amounts of white adipocytes, resulting in an increased susceptibility to COVID-19 infection. Excess pericardial fat found in obesity increases inflammatory cytokines near the heart, in a similar manner to the inflammatory cytokine storm seen in COVID-19 infection. This results in a greater incidence of cardiomyopathy and subsequent heart failure in obese patients following COVID-19 infection, as represented in Figure 7. Obesity is associated with increased risk for cardiovascular disease and heart failure and this risk is further exacerbated by COVID-19 infection. Heart failure is a state of impaired cardiac function that is secondary to many etiologies.

### 1.8. Heart Failure and the Significance of Pericardial Adipose Tissue Proximity to the Heart

The American Heart Association (AHA) classified cardiomyopathy as a disease of the myocardium associated with mechanical or electrical dysfunction that often progresses to heart failure [91]. Cardiomyopathy includes dilated, hypertrophic, and restrictive patterns [92] that can range from minor impairment of cardiac myocytes to complete heart failure [92]. According to the AHA, heart failure is defined as “a complex clinical syndrome that results from any structural or functional impairment of ventricular filling or ejection of blood” [93]. A wide range of factors can result in HF including coronary artery disease (CAD), hypertension, cardiomyopathy, atrial fibrillation, or HF due to obesity. Obesity alone is an independent risk factor for hypertension, CAD, and increased risk for HF, as well as increased morbidity and mortality [94,95,96]. Animal studies have shown that obesity-induced alterations in myocardial lipid metabolism lead to the accumulation of various lipid intermediates that are closely linked to the development of ventricular dysfunction [97]. These pathophysiological changes result in oxidative stress, fibrosis, diastolic dysfunction, and subsequent systolic heart failure [98]. Additionally, obesity and an increased in BMI is associated with increased oxidative stress and LV remodeling [99]. Adipose tissue in general is known to play a significant role in the pathophysiology of patients with heart failure [13,79,100,101,102].

Several studies addressed the significance of epicardial fat and co-morbidities including heart failure with preserved or reduced ejection fraction [36,103]. The adverse effects seen in the presence of excess pericardial fat in obesity are further exacerbated by COVID-19 infection (reviewed in [7]). Studies show that heart failure patients had higher levels of epicardial fat, an indication of cardiovascular risk [13,36,103,104], further confirming the correlation between pericardial fat and heart failure in humans and mice [13]. Pericardial fat increases the onset of cardiovascular disease and may be the cause of increased triglycerides in patients with coronary artery diseases [36,103,105]. Studies showed that pericardial thickness was found to be much greater in patients with chronic atrial fibrillation has on the heart [106]. Further indicating that pericardial fat and the increase expression of inflammatory cytokines adjacent to the heart will impair heart function and intervention with antioxidants attenuated effects of inflammatory cytokines [13,100,107,108]. Excess pericardial may be associated with impaired lung function. Although the mechanism is not entirely understood, pericardial fat was independently associated with restrictive lung patterns in middle-aged adults [109]. The pathway linking pericardial and pulmonary anomalies may also play a role in the detrimental effects that are seen in both the heart and lungs following COVID-19 infection. As previously discussed, COVID-19 invades organs which express high levels of ACE-2 receptor such as the heart, lungs, and liver. Obesity is strongly associated with increased cardiovascular risk and mortality. This increased risk is due to the hyperplasia and hypertrophy of white adipocytes [110].

**Figure 8:**
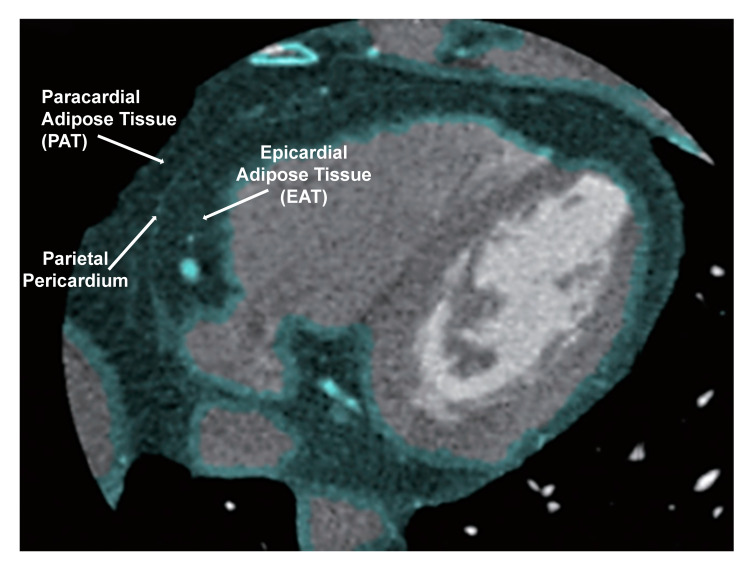
Figures show proximity of Pericardial fat adipose tissue to the heart. Figure also identifies paracardial adipose tissue. Paracardial adipose tissue includes both Pericardial fat and Paracardial adipose tissue.

Cardiovascular risk is not only linked to the overall quantity of adipose tissue, but also to the location of the adipose tissue near the heart [36]. Adipocytes are found in various locations in the human body, including epicardial, paracardial, and pericardial fat which surrounds the heart Figure 8 [111]. Epicardial adipose tissue is essentially the fat depot located directly on the heart and is associated with alterations to cardiac function [106]. Patients with increased visceral adipose tissue have increased epicardial fat volume [112]. Pericardial fat is found directly adjacent to the myocardium with no barrier of fascia separating the two tissues [111]. Under normal states of low oxidative stress, pericardial fat serves an anti-inflammatory function [113]. Pericardial fat secretes factors that regulate physiologic processes in the heart [114]. In patients who are more susceptible to heart failure, such as obese patients, the systemic inflammation that results from COVID-19 infection may further increase the risk for heart failure. As discussed previously, NaKtide, which targets the NAKL may be useful targets to reduce the inflammatory cytokine storm seen in COVID-19 infection. Targeting these cytokines may be an important target for therapy to reduce inflammation in COVID 19 and subsequent effects on the heart. Moreover, increased epicardial adipose tissue alone may be an indicator of increased cardiovascular risk in females [104,115]. All the above observations indicate that pericardial fat conveys the adverse effects of systemic inflammation directly to the heart and sensitivity to COVID. Thus, targeting pericardial fat for treatment options to reduce the risk of heart failure needs to be explored further. It appears that induction of anti-inflammatory and antioxidative agents such as NaKtide may ameliorate the cytokine storm and improve the adverse effects on the myocardium that are exacerbated by COVID-19 infection.

### 1.9. White Adipocytes Increase Inflammation Near the Heart

**Figure 9:**
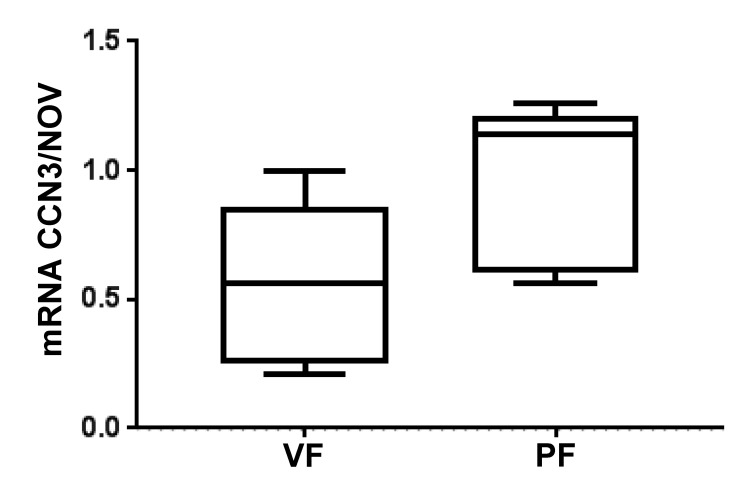
NOV mRNA expression in pericardial fat vs. visceral fat. NOV expression is increased in pericardial fat.

Obesity is associated with adverse remodeling of adipose tissue [116]. Development of heart failure seems to be caused by increased proinflammatory cytokines and reduced cytoprotective autacoids [106]. Excess epicardial and paracardial fat found in obesity are major sources of inflammation which can lead to adverse cardiac remodeling [104]. Dysfunctional adipose tissue releases proinflammatory adipokines that have negative effects on the cardiovascular system [117]. The inflammatory cytokines associated with pericardial fat and obesity include NOV, TNFα, IL-4, and IL-6 [13,67,68]. NOV is associated with increased inflammation and tissue damage and reduction in ejection fraction [13,68,118] and reduction of NOV by increased levels of PGC1-HO-1 contribute to a reversal of heart failure in obesity-Induced diabetic cardiomyopathy [67,68,119]. NOV, in obesity, may potentiate adipocyte hyperplasia, further increasing inflammation Figure 9. Reduction of NOV decreases fat mass and proinflammatory cytokines in adipose tissue of obese mice and ablation of HO-1 increase NOV [120]. A strong association exists between inflammation and NOV in pericardial fat compared visceral fat (Figure 9) and decreases infraction shortening [87,89,121]. Thus, NaKtide with antioxidants ability may represent a therapeutic target to treat the cytokine storm in COVID-19 patients.

### 1.10. White Adipocytes Have Fewer Mitochondria and Reduced Cytoprotection

Obesity increases the volume of pericardial fat followed by inflammation that converts brown fat to white fat along with mitochondrial destruction. This negatively affects cardiovascular risk in obesity. White fat is proinflammatory and has lower levels of mitochondria compared to brown fat. Brown fat can prevent ROS formation through uncoupling mechanisms in the mitochondria by thermogenesis. This allows for the degradation of the electrochemical gradient generated in the mitochondria during oxidative phosphorylation. This process occurs in brown fat and reduces ROS formation. This uncoupling process does not occur in obesity because there is a greater number of white adipocytes which contain fewer mitochondria. The result is greater ROS in obese patients. Similarly, the expression of uncoupling gene UCP1 and mitochondrial dynamics-related mitofusin gene MFN2 are reduced in epicardial fat compared with visceral fat [13].

**Figure 10:**
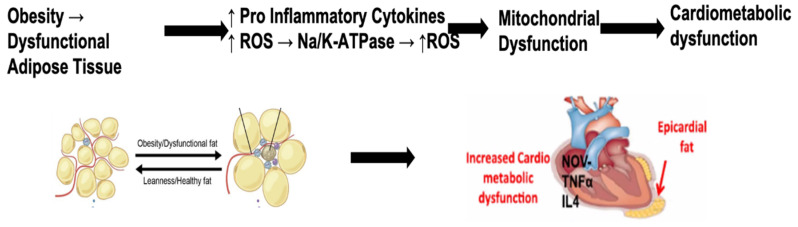
Schematic depicts a pathway leading to obesity induced cardiometabolic dysfunction. This pathway is further exacerbated by COVID-19 infection.

Morbid obesity causes increased pericardial thickness which has a direct association with increased left ventricular mass and decreased left ventricular function [99,101]. This increased pericardial thickness is also associated with impaired fractional shortening [101,104]. Cardiac function measured with echocardiography was found to be impaired in obese mice compared to lean mice. This reduced function was determined by analyzing left ventricular fractional shortening [13]. Pericardial fat is associated with cardiomyopathy and cardiac remodeling [14,122]. Figure 10 represents fat accumulation and inflammation adjacent to the epicardium, resulting in increased ROS with adverse effects on cardiac structure and function [123]. Increased ROS can cause cell death and release highly reactive free radicals that induce adverse cardiac remodeling [124,125].

As shown on Figure 10, NaKtide is a potential target for attenuation of left ventricular dysfunction caused by obesity. Naktides increased PGC1α that is critical for restoring left ventricular function [13]. Increases in PGC1α are associated with a reduction in fat deposition, which results in a reduction of adipose-mediated increases major inflammatory adipokines and NOV expression [13,68,119]. This decrease in NOV is associated with a reduction of other inflammatory adipokines and may have a direct effect on left ventricular fractional shortening via increase of brown stem cells [126,127]. The importance of brown adipocyte level in adipose tissue stems from the key role these cells have in maintenance of mitochondrial function, anti-inflammation and anti-obesity, thermogenesis [128] and production and release of signaling molecules. Gao et al.l [129] and others [130] demonstrated that high fat diet (HFD) intake led to the reduction of AMPK, UCP1, PGC1, and Foxp1 [131] with a concomitant decrease in the number of brown adipocytes. In contrast, an increase in SIRT3 [131] and PGC1 augment the number of brown adipocytes and improve heart function [13,132] Additionally, expansion of white adipocytes increases infiltration of inflammatory cells such as M1-like macrophages and CD8+ cells, with impairment of the molecular network that participates in reprograming white adipocytes to the brown phenotype. Levels of brown adipocytes can be influenced by caloric restriction, or intake of agriculture-derived Chinese medicine Jinlida granulate and Cinnamam cassia extract [133,134] and nutrients such as pomegranates, Milk thistle, Cumen, and black seed oil [44,45,135], all via an increase in mitochondrial function and the signaling network involving PGC1, SIRT1, and fibroblast growth factor 21 (FGF21), as well as energy expenditure. Targeting adipose tissue with NaKtide will result in a sustained number of brown-like adipocytes that allow to combat obesity by increased thermogenesis, fat burning and amelioration of the cytokine storm. These factors suggested that NaKtide administration may reduce the severe inflammation that is seen in obese patients following COVID-19 infection. This may assist with restoring left ventricular function.

## 2. Conclusions

This review summarizes findings supporting the notion that chronic obesity results in a state of systemic inflammation that has many downstream effects on distal organ function. These effects include increased ROS production, low baseline levels of cytoprotective autocoids, uncoupling of mitochondrial enzymes, and increased Src activity due to carbonylation of the Na/K-ATPase α1 subunit with cytokines storm and heart failure. These effects are further exacerbated by SARS-CoV-2 which induces a cytokine storm. Thus, there is an increased risk of renal dysfunction and subsequent heart failure in COVID-19 patients with obesity. Until a vaccine for COVID-19 is found, we suggest that a combination of natural antioxidants and pNaKtide can block NKAL-derived can sever ROS and ultimately ameliorate the production of the cytokine storm and heart failure.
